# Metachronous Small Bowel and Sigmoid Volvulus in an Elderly Male: A Case Report

**DOI:** 10.7759/cureus.92306

**Published:** 2025-09-14

**Authors:** Yoichi Miyaoka, Shingo Shimada, Yuichi Yoshida, Ryoji Yokoyama

**Affiliations:** 1 General Surgery, Abashiri-Kosei General Hospital, Abashiri, JPN; 2 Gastroenterological Surgery I, Hokkaido University Graduate School of Medicine, Sapporo, JPN

**Keywords:** metachronous volvulus, sigmoid colectomy, sigmoid volvulus, small-bowel volvulus, whirl sign

## Abstract

Volvulus, torsion of the intestine causing luminal obstruction and vascular compromise, is a surgical emergency most commonly involving the sigmoid colon in adults; small-bowel volvulus (SBV) is less frequent in developed countries. Metachronous volvulus arising in different intestinal segments within the same individual is exceptionally rare. We report an elderly male who underwent emergency surgery for ileal volvulus and, several years later, developed sigmoid volvulus (SB) requiring colectomy. This case highlights a possible shared predisposition (elongated/mobile bowel on a narrow mesenteric base and postsurgical adhesions), underscores the diagnostic value of computed tomography, and supports early elective sigmoidectomy after successful endoscopic detorsion to prevent recurrence and ischemic complications.

## Introduction

Volvulus, defined as a twisting of the bowel loop around its mesenteric axis, can cause acute intestinal obstruction and compromise vascular perfusion, constituting a surgical emergency [1]. Among adults, sigmoid volvulus (SV) is the most common form of colonic volvulus, accounting for approximately 60-80% of cases, and represents 10-15% of all large-bowel obstructions in Western countries [2]. Known predisposing factors include chronic constipation, a high-fiber diet, laxative overuse, neuropsychiatric illness, chronic bedridden status, and anatomical features, such as a redundant and mobile sigmoid colon (dolichosigmoid) with a narrow mesenteric base [3]. In contrast, small-bowel volvulus (SBV) is relatively rare in developed nations, with an estimated incidence of 1.7-5.7 per 100,000 person-years in North America and Europe [4]. Primary SBV, which occurs without an underlying anatomical or pathological lesion, is particularly uncommon in elderly individuals. Secondary SBV is more frequently observed and is usually related to postoperative adhesions, congenital malrotation, tumors, or Meckel’s diverticulum [4,5]. Metachronous volvulus involving different intestinal segments is very rare, particularly in elderly patients, and presents unique diagnostic and therapeutic challenges. While a few cases of cecal volvulus following SV or simultaneous ileosigmoid knotting have been reported [6,7], these sequential presentations underscore the clinical significance of recognizing and managing predisposition to torsion at multiple sites. We here report a case of SV that developed several years after surgical treatment for SBV. We also discuss diagnostic and therapeutic considerations for such sequential presentations. Globally, an estimated five billion people lack access to safe, affordable surgical and anesthesia care, underscoring the need for timely recognition and management of surgical emergencies such as volvulus [8].

## Case presentation

An 81-year-old man presented to the emergency department with abdominal distension and severe abdominal pain. His past medical history included old myocardial infarction, lumbar spinal canal stenosis, chronic kidney disease, and pulmonary emphysema.

**Figure 1:**
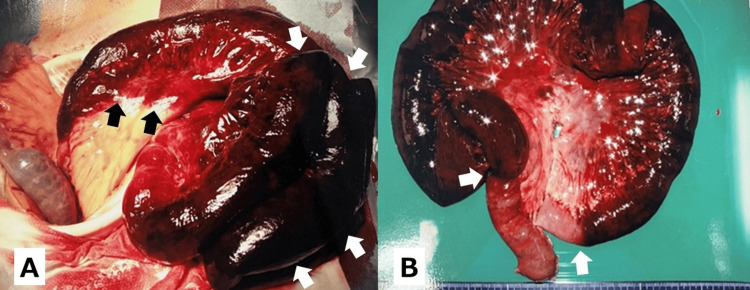
Primary small-bowel volvulus at age 70. (A) Intraoperative view showing tense, dusky-black ileal loops consistent with strangulated midgut volvulus (white arrows). The black arrows mark the interface between the abnormally reddened (hyperemic/congested) mesentery and the adjacent normal mesentery. (B) Resected specimen showing dusky-black discoloration and serosal hemorrhage consistent with transmural hemorrhagic infarction. The white arrows indicate the boundary of the discolored segment and a constriction (strangulation groove) on the bowel. The gangrenous bowel extended from approximately 190-320 cm distal to the ligament of Treitz.

At the age of 70, the patient, who had no prior history of abdominal surgery, presented with acute abdominal pain and was diagnosed with strangulated small-bowel obstruction due to ileal volvulus. Emergency laparotomy revealed a clockwise torsion of the ileum around its mesenteric axis, resulting in ischemia without any identifiable obstructing band or lesion. In addition to the torsion, there was marked mesenteric congestion and a small amount of serosanguinous peritoneal fluid. A gangrenous segment extending from 190 cm to 320 cm distal to the ligament of Treitz was resected, and a primary anastomosis was performed (Figure 1). Histopathological examination confirmed transmural hemorrhagic infarction in the resected bowel.

Three years later, he developed SV, which was initially managed non-operatively with endoscopic detorsion. He subsequently experienced multiple recurrent episodes, all managed similarly with colonoscopic reduction. Despite repeated recommendations for elective surgery, the patient consistently declined operative intervention.

**Figure 2:**
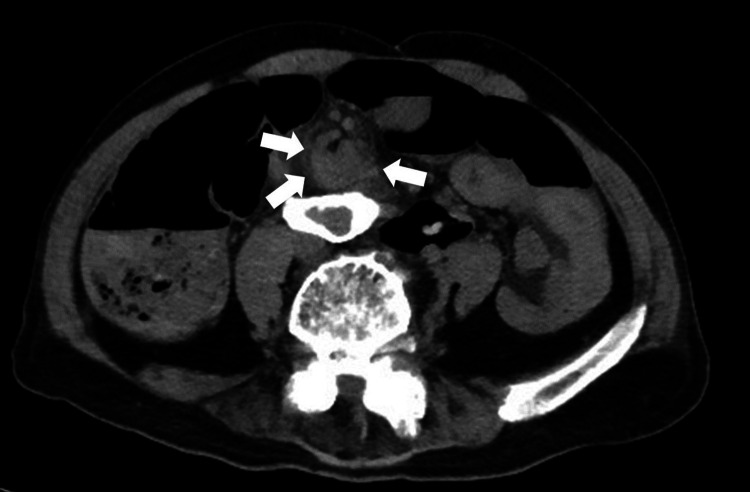
Recurrent sigmoid volvulus—axial non-contrast CT. Axial non-contrast CT demonstrates a mesenteric “whirl sign” centered on the sigmoid mesentery (white arrows) with marked upstream colonic dilatation, compatible with sigmoid volvulus.

Approximately one year after his last recurrence, he remained stable without further volvulus episodes until he presented again with acute abdominal distension and pain. CT imaging revealed marked colonic dilation and a classic “whirl sign” at the sigmoid colon (Figure 2), confirming recurrent SV. Endoscopic reduction was successfully performed, and the patient was elected to proceed with surgery at that time.

**Figure 3:**
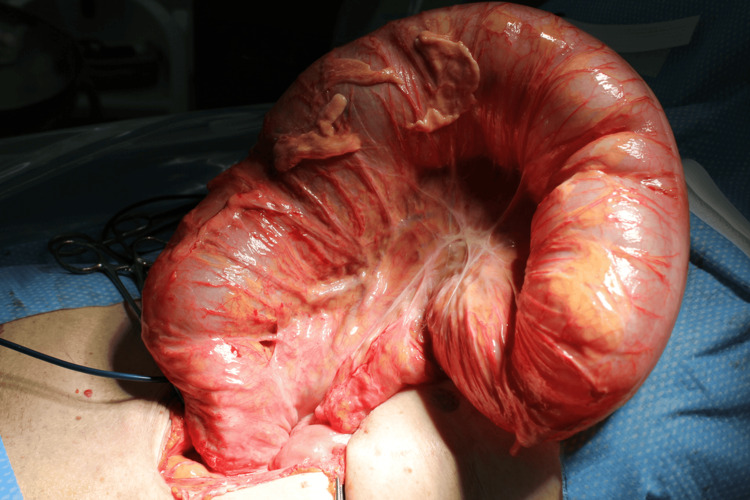
Definitive surgery for recurrent sigmoid volvulus. Intraoperative photograph showing a markedly dilated, redundant sigmoid colon exteriorized through a mini-laparotomy after conversion from laparoscopy due to dense adhesions; sigmoid colectomy with primary anastomosis was performed.

A laparoscopic approach was attempted with a 2 cm umbilical incision for the camera port. Upon insufflation and inspection, dense adhesions were identified intraoperatively, predominantly between the small bowel, abdominal wall, and sigmoid colon, and these were inferred as being related to the prior small-bowel surgery. The conversion was performed solely because of these dense adhesions, which significantly limited visualization and safe manipulation. The patient remained hemodynamically stable throughout the procedure, and there was no intraoperative concern for bowel injury at the time of conversion. The procedure was converted to a mini-laparotomy through a small midline incision. The dilated sigmoid colon was exteriorized, resected, and a primary anastomosis was performed (Figure 3). Operative time was 77 minutes, and the estimated blood loss was 30 mL, which was within the expected range for such a case.

The postoperative course was notable for transient paralytic ileus, but oral intake was resumed by postoperative day seven. He recovered without major complications and was discharged on postoperative day 14. At one-year follow-up, there have been no signs of recurrence.

## Discussion

SV and SBV are individually uncommon but well-recognized surgical emergencies. Their sequential, metachronous occurrence in a single patient, separated by years and requiring two major resections, is exceptionally uncommon and has been reported only sporadically in the literature. In particular, elderly patients represent an unusual subgroup in which such sequential presentations are even less frequently described. While isolated cases of metachronous volvulus involving different colonic segments and occasional ileosigmoid knotting have been described, these remain anecdotal and underscore the rarity of our patient’s presentation [1,2,6,7]. Several factors may contribute to a shared predisposition. Risk factors well supported by prior studies include anatomical vulnerabilities, such as a redundant/mobile sigmoid colon with a narrow mesenteric base and chronic constipation, which are recognized contributors to SV [2-5]. In contrast, case-specific factors inferred in our patient included dense postoperative adhesions - likely secondary to the prior small-bowel resection - that altered intra-abdominal anatomy, and age-related dysmotility that may predispose to recurrent overdistension. Differentiating between evidence-based and case-specific elements helps clarify why this patient developed sequential volvulus in two distinct intestinal segments. For uncomplicated SV, urgent endoscopic detorsion is the recommended initial therapy; however, recurrence is common, and contemporary guidance supports definitive sigmoid resection during the index admission or soon thereafter in medically fit patients to prevent progression to gangrene or perforation [3,9,10]. It is also generally recognized that elective sigmoidectomy following successful detorsion is associated with substantially lower mortality and morbidity compared with emergent surgery performed for gangrenous volvulus, further supporting the role of timely elective surgery in suitable patients. Elective sigmoidectomy carries substantially lower mortality than emergent colectomy for gangrenous SV and provides durable prevention of recurrence; minimally invasive resection is often feasible, although prior laparotomy and dense adhesions may necessitate conversion, which should be anticipated and discussed preoperatively [9,10]. Cross-sectional computed tomography is the diagnostic modality of choice to confirm volvulus, localize the involved segment, and assess for ischemia or perforation [4,9]; in particular, the mesenteric “whirl sign” on CT is a reliable radiographic marker that supports timely decision-making [11]. Taken together, a history of SBV should heighten vigilance for subsequent colonic volvulus, each successful detorsion should prompt candid counseling about definitive surgery, and timely elective sigmoidectomy after recurrence offers the most reliable protection against further morbidity and mortality.

## Conclusions

This case highlights the rare occurrence of metachronous volvulus involving both the small bowel and sigmoid colon in the same patient. Surgeons should be aware that patients with a history of SBV may later develop colonic volvulus, particularly in the sigmoid colon, due to anatomical predisposition and acquired redundancy. Careful follow-up and early recognition of recurrent or new-onset volvulus are essential to avoid life-threatening complications.
